# Insertable cardiac monitor‐guided early intervention to reduce atrial fibrillation burden following catheter ablation: Study design and clinical protocol (ICM‐REDUCE‐AF trial)

**DOI:** 10.1111/anec.13043

**Published:** 2023-01-31

**Authors:** Sinan S. Tankut, David T. Huang, Wojciech Zareba, Mehmet K. Aktas, Spencer Z. Rosero, Jonathan Steinberg, Jennifer Henchen, Valentina Kutyifa, Robert L. Strawderman, Ilan Goldenberg

**Affiliations:** ^1^ Division of Cardiology University of Rochester Medical Center Rochester New York USA; ^2^ Clinical Cardiovascular Research Center University of Rochester Medical Center Rochester New York USA; ^3^ Summit Medical Group Short Hills New Jersey USA

**Keywords:** atrial fibrillation, catheter ablation, insertable cardiac monitor, remote monitoring

## Abstract

**Background:**

Percutaneous catheter ablation (CA) to achieve pulmonary vein isolation is an effective treatment for drug‐refractory paroxysmal and persistent atrial fibrillation (AF). However, recurrence rates after a single AF ablation procedure remain elevated. Conventional management after CA ablation has mostly been based on clinical AF recurrence. However, continuous recordings with insertable cardiac monitors (ICMs) and patient‐triggered mobile app transmissions post‐CA can now be used to detect early recurrences of subclinical AF (SCAF). We hypothesize that early intervention following CA based on personalized ICM data can prevent the substrate progression that promotes the onset and maintenance of atrial arrhythmias.

**Methods:**

This is a randomized, double‐blind (to SCAF data), single‐tertiary center clinical trial in which 120 patients with drug‐refractory paroxysmal or persistent AF are planned to undergo CA with an ICM. Randomization will be to *an intervention arm* (*n* = 60) consisting of ICM‐guided early intervention based on SCAF and patient‐triggered mobile app transmissions versus *a control arm* (*n* = 60) consisting of a standard intervention protocol based on clinical AF recurrence validated by the ICM. Primary endpoint is AF burden, which will be assessed from ICMs at 15 months post‐AF ablation. Secondary endpoints include healthcare utilization, functional capacity, and quality of life.

**Conclusion:**

We believe that ICM‐guided early intervention will provide a novel, personalized approach to post‐AF ablation management that will result in a significant reduction in AF burden, healthcare utilization, and improvements in functional capacity and quality of life.

## Graphical abstract

This is a randomized, double‐blind, single‐tertiary center clinical trial in which 120 patients with drug‐refractory paroxysmal or persistent atrial fibrillation (AF) are planned to undergo catheter ablation with an insertable cardiac monitor (ICM). Randomization will be to an intervention arm (*n* = 60) consisting of ICM‐guided early intervention based on subclinical AF and patient‐triggered mobile app transmissions versus a control arm (*n* = 60) consisting of a standard intervention protocol based on clinical AF recurrence validated by the ICM. We believe that ICM‐guided early intervention will provide a novel, personalized approach to post‐AF ablation management that will result in a significant reduction in AF burden, healthcare utilization, and improvements in functional capacity and quality of life.

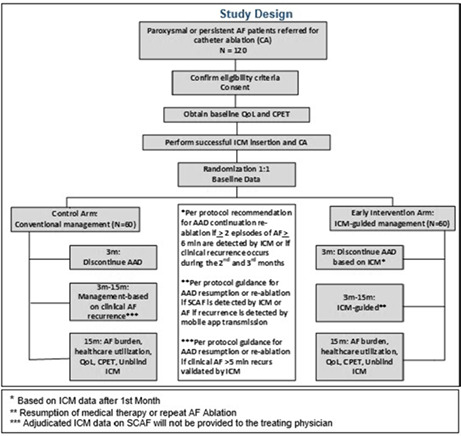

## 1 INTRODUCTION

Atrial fibrillation (AF) is a major public health concern in the United States and is associated with significant morbidity, mortality, and healthcare costs. Catheter ablation (CA), mostly in the form of pulmonary vein isolation (PVI), has been shown to be effective in restoring and maintaining sinus rhythm (January et al., 2014, 2019; Kim et al., 2011; Kirchhof et al., 2016).

Recurrence rates of AF after a single CA procedure continue to be a concern, and many patients require more than one procedure to achieve symptom control (Calkins et al., 2009). While recurrences of AF after CA are common during the first 3 months (“blanking period”), they are associated with an increased risk of procedural failure (Liang et al., 2016). The most common reason for AF recurrence after CA is a reconnection of electrically active pulmonary veins. This results in continued AF‐induced atrial remodeling that facilitates further progression of AF after the ablation procedure (Atul et al., 2005; Callans et al., 2004; Gerstenfeld et al., 2003; Nanthakumar et al., 2004). In the CABANA study (Effect of Catheter Ablation vs. Antiarrhythmic Drug therapy on mortality, stroke, bleeding, and cardiac arrest among AF patients), detection of clinical recurrence of paroxysmal or persistent AF using the study's electrocardiogram event recorder approached 40% at 1‐year (Packer et al., 2019). There are now increasing data suggesting that early intervention for AF recurrence may improve long‐term outcomes and reduce AF burden following CA.

The clinical significance of subclinical atrial fibrillation (SCAF) detected by insertable cardiac monitors (ICM), and the effectiveness of early intervention following ablation remains controversial. To date, studies have relied on conventional symptom reporting and discontinuous monitoring to detect recurrent AF after CA. However, recent data suggest that symptoms may be an underestimate of postablation AF burden, and nearly 40% of patients experiencing recurrence after AF ablation remain asymptomatic (Pedrote et al., 2013; Tondo et al., 2014; Verma et al., 2013; Wechselberger et al., 2018). In the DISCERN AF study (Discerning the incidence of symptomatic and asymptomatic episodes of AF before and after CA), the proportion of AF episodes that were asymptomatic (SCAF), increased from 52% before to 79% after ablation (*p* = .002) and the postablation state was shown to be the strongest predictor of SCAF (Verma et al., 2013). Therefore, early intervention following the detection of SCAF by an ICM may be important to prevent substrate progression. The ICM‐REDUCE‐AF study is designed to test the hypothesis that an ICM‐guided early intervention strategy following CA will lead to a greater reduction in AF burden, healthcare utilization, and improvement in functional capacity.

## 2 METHODS

### 2.1 Objectives

The primary objective of the ICM‐REDUCE‐AF study is to determine whether an ICM‐guided early intervention strategy following CA of drug‐refractory paroxysmal or persistent AF will lead to a greater reduction in AF burden.

The secondary objectives of this trial, in a prioritized order are as follows:

1. Evaluate whether an ICM‐guided early intervention strategy following CA of drug‐refractory paroxysmal or persistent AF is associated with a significant reduction in healthcare utilization; defined as unplanned hospitalizations, emergency department (ED) visits, office visits, or cardioversions.
2. Determine whether an ICM‐guided early intervention strategy following CA of drug‐refractory paroxysmal or persistent AF is associated with improvements in functional capacity (assessed by peak VO_2_ that is derived from cardiopulmonary exercise test [CPET], activity, and quality of life (QoL) assessed via the Atrial Fibrillation Effect on Quality‐of‐Life [AFEQT] Questionnaire) compared with conventional management.

### 2.2 Design

**FIGURE 1:**
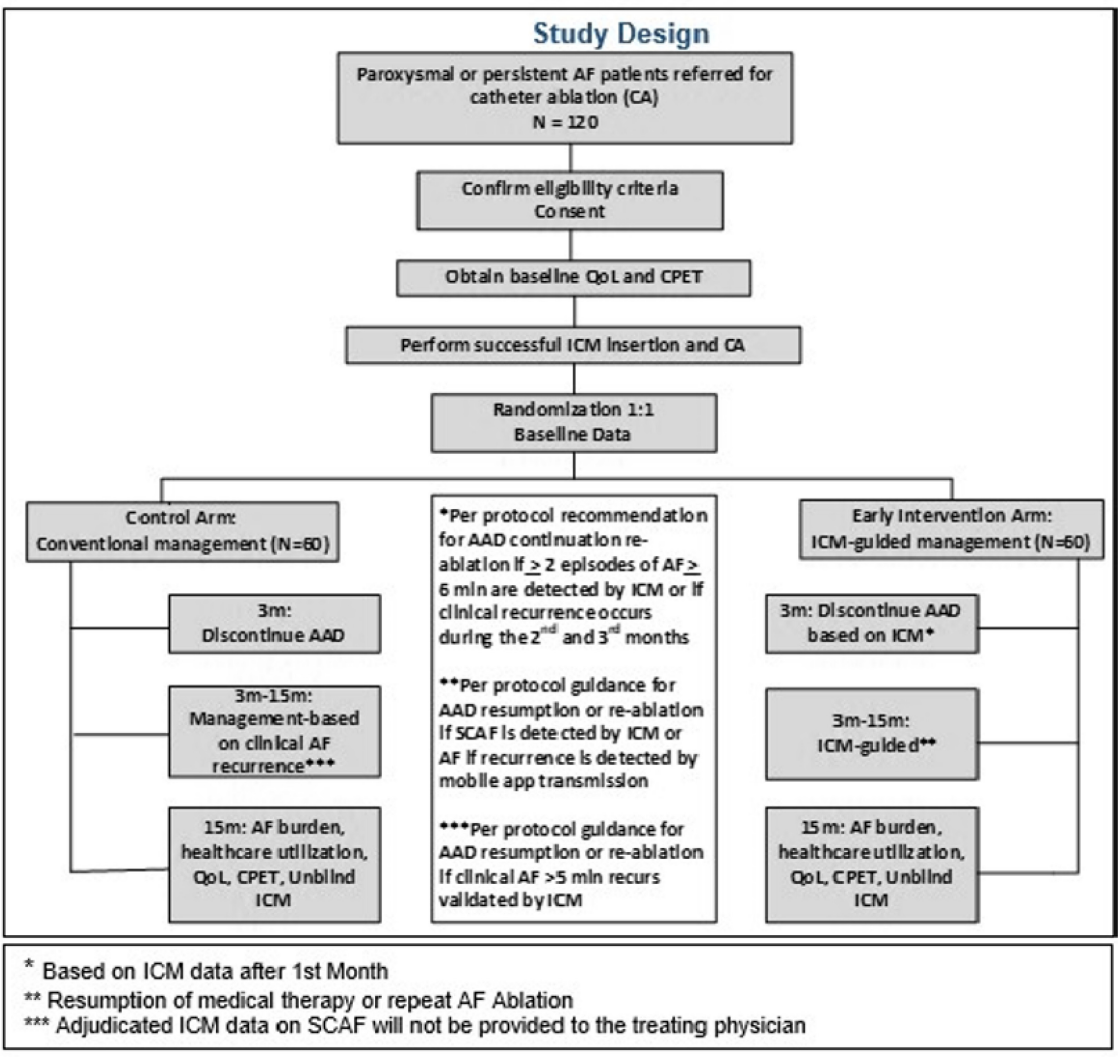
Study design

In this study, subjects with drug‐refractory paroxysmal or persistent AF will be randomized to CA with conventional management based on clinical AF recurrence after the CA procedure (control arm) or to an ICM‐guided early intervention strategy (early intervention arm) (Figure 1). This will be a prospective, single‐tertiary center of a large healthcare system, double‐blind (to SCAF data), randomized clinical trial enrolling 120 patients with drug‐refractory paroxysmal or persistent typical AF who have been referred for a CA with ICM monitoring based on conventional clinical indications.

### 2.3 Study population

The patient population for this trial consists of patients with drug‐refractory paroxysmal or persistent AF referred for CA based on conventional clinical indications.

Patients will be stratified in a 1:1 ratio by the type of AF (i.e., paroxysmal vs. persistent AF). The study protocol was approved by the URMC ethics committee and conducted in compliance with standard institutional operating procedures and the Declaration of Helsinki. All patients enrolled in the study will provide written informed consent.

#### 2.3.1 Eligibility

- Patients with *paroxysmal AF* refractory or intolerant to at least one antiarrhythmic agent, according to current guideline indications for paroxysmal AF ablation (Class I); (January et al., 2014, 2019) or patients with *persistent AF* refractory or intolerant to at least one antiarrhythmic agent, according to current guideline indications for persistent AF ablation (Class IIa) (January et al., 2014, 2019).
- 50 years of age or older
- CHA_2_DS_2_‐VASc ≥2 in men or ≥3 in women at the time of consent
- Undergoing CA for AF for the first time
- ICM indicated for monitoring symptoms after AF ablation as standard of care
- Signed informed consent and willing to comply with the study protocol

#### 2.3.2 Exclusions

- Inability to tolerate any AAD therapy
- Sustained AF lasting more than 3 years
- Left atrial diameter of 60 mm or greater
- Inability to undergo AF CA (e.g., presence of a left atrial thrombus, contraindication to anticoagulation)
- Prior ablation for an atrial arrhythmia, either by catheter or by surgery
- NYHA class IV congestive heart failure
- Patients with an implantable cardiac rhythm device (ICD/CRTD/PPM)
- LV ejection fraction ≤35% if indicated for an ICD
- Coronary revascularization or valve surgery within 3 months prior to consent day
- Prior valve surgery using a mechanical prosthesis
- Life expectancy <1 year for any medical condition
- Unwillingness to comply with all postprocedural follow‐up requirements and to sign informed consent
- Patients with metabolic derangements (e.g., renal/hepatic failure, electrolyte disturbance, etc.), prohibiting electrophysiology study and ablation or AAD medical therapy (e.g., dofetilide, sotalol, or amiodarone, etc.)
- Patients with an intracardiac thrombus
- Pregnancy or nursing
- Contraindication to anticoagulant therapy

### 2.4 Study design

The design of the study and the schedule of activities are presented in Figure 1 and Table 1, respectively. This will be a prospective, single‐tertiary center (of a large Healthcare System in Upstate New York), double‐blind (to SCAF data), randomized clinical trial enrolling 120 patients with drug‐refractory paroxysmal or persistent typical AF who have been referred for a CA with ICM monitoring based on conventional clinical indications.

**TABLE 1:** Scheduled activities

Reportable data items	Screening/enrollment/randomization	Follow‐up visits				
	Visit 1	Tele‐visit 2	Tele‐visit 3	Tele‐visit 4	Tele‐visit 5	Clinic visit 6
		1 month after randomization +/− 14 days	3 months after randomization +/− 30 days	6 months after randomization +/− 30 days	12 months after randomization +/− 30 days	15 months after randomization +/− 30 days
Inclusion/Exclusion	√					
Informed consent	√					
Demographics	√					
Medical History	√					
Physical exam	√					√
Cardiac Medications	√	√	√	√	√	√
NYHA class	√	√	√	√	√	√
12‐lead ECG (most recent)^a^	√	√	√	√	√	√
Echo (most recent)^a^	√					
AFEQT^b^	*√*					*√*
CPET	√					√
AF ablation^a^	√					
ICM insertion^a^	*√*					
ICM data collection^c^		*√*	*√*	*√*	√	√
Repeat procedures/change in medications		√	√	√	√	√
Healthcare utilization^d^		√	√	√	√	√
Adverse Events^e^		√	√	√	√	√
Protocol deviations		√	√	√	√	√
Subject status		√	√	√	√	√

^a^ Standard of care procedures.

^b^ QualiTy‐of‐life (AFEQT) questionnaire will be used to assess QoL.

^c^ Interrogation committee will meet on a monthly basis.

^d^ defined as unplanned hospitalizations, ED visits, cardioversions, and unplanned office visits (beyond those designated as study visits).

^e^ defined as unplanned hospitalizations, ED visits, and cardioversions.

#### 2.4.1 Recruitment

Recruitment will occur at the University of Rochester Healthcare System, comprising three hospitals and associated outpatient clinics in Upstate New York. Screening for enrollment will be conducted in compliance with HIPPA requirements. Subjects who meet the eligibility criteria and do not have any exclusions will be recruited by the clinical electrophysiology groups associated with URMC.

#### 2.4.2 Consent

The study, including the entailed procedure's possible benefits and risk, will be discussed with the patient. A brochure which contains detailed information in regard to the study will be provided to all subjects. The patient will be required to sign a consent for participation in ICM‐REDUCE‐AF.

#### 2.4.3 Randomization

Study subjects will be randomized 1:1 to conventional management based on clinical AF recurrence after the CA procedure (control arm) or to an ICM‐guided early intervention strategy (early intervention arm), with prespecified arrhythmic, clinical, functional, and QoL endpoints.

#### 2.4.4 Baseline evaluation

After enrollment and randomization, baseline testing will be performed including a clinical history, physical exam, 12–lead ECG, echocardiogram, CPET, and QoL assessed by the AFEQT questionnaire. CPET will be performed in the rhythm (sinus rhythm vs. AF) the patient is presenting with day of their testing.

#### 2.4.5 Follow‐up

Following baseline testing, patients will undergo AF ablation with ICM insertion per standard clinical practice and will be followed over 15 months (comprising the 3‐month blanking period and the subsequent 12 months of follow‐up for AF burden assessment). Subsequent study visits will be virtual (through phone contact and/or videoconferencing) in which: (a) remote ICM interrogations will be transmitted to the Data Coordination Center (DCC) by the site research coordinator; (b) follow‐up data on subject status and healthcare utilization/adverse events will be obtained; (c) AF management will be adjusted based on the prespecified study protocol supervised by the DCC. CPET, and QoL testing will be repeated at the 15‐month follow‐up visit.

#### 2.4.6 Post‐AF ablation management in the early intervention arm

**FIGURE 2:**
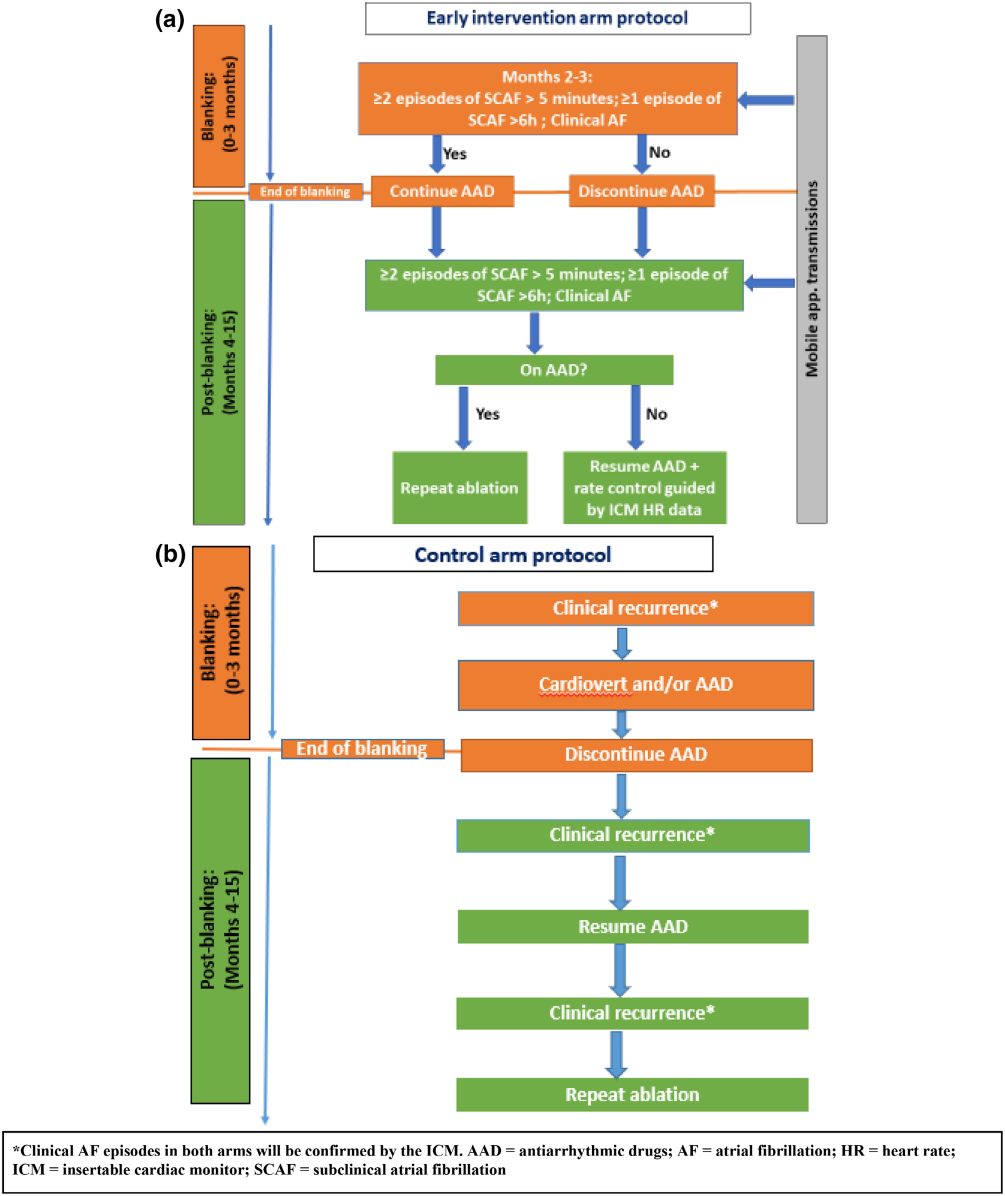
Study protocol. *Clinical AF episodes in both arms will be confirmed by the ICM. AAD, antiarrhythmic drugs; AF, atrial fibrillation; HR, heart rate; ICM, insertable cardiac monitor; SCAF, subclinical atrial fibrillation

In the early intervention arm, the DCC study manager will transmit electronic notifications to the site principal investigator (PI) and research coordinator on the recommended treatment change (i.e., antiarrhythmic drug resumption and/or repeat ablation) based on adjudicated detection of SCAF and patient‐triggered mobile app AF transmissions. Based on data on the duration of AF that is associated with increased risk of progression, SCAF requiring early intervention will be defined in the present study as ≥2 episodes lasting >5 min but <6 h or one episode lasting >6 h. Physicians and patients will be blinded to ICM interrogations. If SCAF is detected by ICM adjudication or if a patient‐triggered mobile app transmission is confirmed as AF at the DCC, the study manager at the DCC will send electronic notifications to the site PI and research coordinator on the protocol recommendations (Figure 2a).

*Management during the blanking period*: Similar to the control arm, medical therapy with AAD in the intervention arm will continue for 3 months after the index CA procedure arm. Symptomatic AF recurrence during the blanking period will be treated with cardioversions. If SCAF is detected by the ICM or symptomatic AF recurrence occurs after one‐month postablation (i.e., during the second or third months), AAD therapy will not be discontinued at the 3 months per protocol. AF recurrence during the blanking period will not be an indication for repeat ablation.

*Management after the blanking period*: If SCAF recurrence is detected by ICM adjudication or if symptoms resulting in a patient‐triggered mobile app transmission are confirmed as AF, the study manager at the DCC will send electronic notifications to the site PI and research coordinator on the protocol recommendation for AAD therapy resumption (if discontinued at 3 months) or for referral for reablation (if already on AAD therapy). Clinical AF recurrence in the early intervention arm will be managed using the same protocol as in the control arm (Figure 2a).

#### 2.4.7 Post‐AF ablation management in the control arm

Management in the control arm will be protocoled based on clinical AF recurrences (Figure 2b). Physicians and patients will be blinded to SCAF detected by the ICM but will be provided by the DCC upon request with full ICM information on any clinical episodes.

*Management during the blanking period (≤3 months)*: Medical therapy with AAD will continue for 3 months in all study patients after the index CA procedure. Clinical recurrences during the blanking period will be treated with cardioversions and/or AAD intensification. Discontinuation of AAD therapy after the blanking period is recommended for all patients in the control arm.

*Management after the blanking period (>3 months)*: Upon the development of clinical AF recurrence after the blanking period (defined as symptoms resulting in medical contact, confirmed by the ICM as AF episodes > 5 min), treatment with AAD will be resumed. If a second clinical AF recurs despite AAD, repeat ablation is to be performed. The DCC will provide ICM data on clinical episodes upon request by the treating physician, as well as complete data on all ventricular arrhythmic events. Data on patient‐triggered mobile app transmissions will also be provided by the DCC upon request to physicians of patients in the control arm but will only be used to guide management in the early intervention arm. ICMs will be opened to physicians upon study closure to provide long‐term arrhythmia monitoring.

#### 2.4.8 Management common to both arms

Physicians and patients in both arms will be blinded to ICM data on SCAF adjudicated by an independent committee that is comprised of three electrophysiologists at the DCC. ICM data on clinical episodes (defined as symptoms resulting in medical contact) will not be blinded and will be made available by the DCC to physicians in both arms upon request. Protocol recommendations will be mandated in both arms unless deemed contraindicated by the treating physician due to a change in the patient's health status after enrollment. ICM interrogations will be done via an electronic system. The blinded data from the ICM interrogations will be sent to the DCC. From this location, if AF requiring intervention is detected in the intervention arm, the adjudicated data are unblinded, and appropriate treatment recommendations are sent to the site PI and research coordinator. The DCC will provide full data on all other ICM‐adjudicated arrhythmic episodes, including ventricular arrhythmias (regardless of symptoms). ICM data will be opened upon study closure (15 months) to provide physicians access for long‐term arrhythmia monitoring.

### 2.5 Endpoints

The primary endpoint of the trial is AF burden between 3 and 15 months after the index CA procedure, as detected by an ICM, which will be inserted prior to or at the time of AF ablation procedure in all study patients. AF recurrence is defined as any episode of AF lasting >30 s per published consensus statements (Calkins et al., 2017). AF burden will be defined as the mean amount of time spent in AF over the prespecified period of time (excluding short AF episodes of ≤30 s).

#### Secondary endpoints are

1. Healthcare utilization, defined as the cumulative total of hospitalizations, ED visits, cardioversions, and office visits (beyond those designated as study visits Table 1) in each arm from the index CA procedure through 15 months.
2. Functional capacity, as assessed by the gold standard of peak VO_2_, which will be determined from CPET performed at baseline and at 15 months.
3. Functional capacity, as assessed by the average daily count of steps derived from the ICM, between baseline and 15 months.
4. QoL measures using the AFEQT questionnaire obtained at baseline and at 15 months.

#### Tertiary (exploratory) endpoints are

1. Clinically significant (>30 min) atrial arrhythmia (AF, atrial flutter, or atrial tachycardia) as detected and documented by ICM after the performance of the index AF ablation procedure (excluding the initial 3‐month blanking period).
2. Symptomatic AF recurrence (regardless of duration), summarized as number of episodes per patient confirmed as AF by the ICM.
3. Adverse events (more severe components of the healthcare utilization endpoint, comprising unplanned hospitalizations, ED visits, and cardioversions).

### 2.6 Statistical analysis

This two‐arm 1:1 randomized clinical trial will randomize 120 patients to ICM‐guided management post‐CA of AF versus conventional management after the procedure (60 patients per arm). For objective 1 (AF burden), based on prior studies the expected AF burden in the conventional management group is 5.3% + 2.6% in patients with paroxysmal AF and 25% ± 12% in patients with persistent AF (Wechselberger et al., 2018). Utilizing these means and standard deviations to guide the study design, we expect to achieve >90% (89%–99.9%) power to detect a 40% (30%–50%) relative reduction in the rate of AF burden between months 4 and 15 (i.e., 1 year after the blanking period), using a two‐sample, one‐sided 0.05‐level *t*‐test allowing for unequal variances. Similar power (86%–99.9%) is maintained if the AF burden in the persistent AF control group is lower than expected (20%, SD = 10%) and the variances are each inflated by a factor of 10% to reflect the influence of dropout. For objective 2 (healthcare utilization), we expect ICM‐guided management in the intervention arm to be associated with a 30% reduction in healthcare utilization (defined as unplanned hospitalizations, ED visits, office visits, or cardioversions). Expected healthcare utilization is based on prior literature (Ladapo et al., 2012). A sample size of 120 patients, randomized 1:1 to ICM‐guided management versus conventional management would provide >90% power to detect a 30% relative reduction in the rate of healthcare utilization (as defined above) during the 15‐month study period. This power analysis is for a one‐sided, 0.05 level test for a reduction in the rate due to treatment, derived under the following assumptions: that the control group experiences a monthly utilization rate of 2.2 visits with extra‐Poisson variation in the monthly counts (i.e., SD = 1.84 visits per month instead of 1.47); the treatment group experiences a monthly utilization rate of 1.5 visits with extra‐Poisson variation in the monthly counts (i.e., SD = 1.44 visits per month instead of 1.22); the treatment effect is estimated under a quasi‐Poisson generalized linear regression model that allows for extra‐Poisson variation; a Wald test is used to evaluate the treatment effect and uses a robust standard error (SE) estimate, and, a 10% death or loss to follow‐up over the 15‐month study period. A similar approach will be utilized to evaluate the tertiary endpoint of more severe adverse events (comprising unplanned hospitalizations, ED visits, and cardioversions). Based on URMC and published data (Packer et al., 2019), the death rate at 15 months following CA for AF is low (1%–2%) and will, therefore, not be used for endpoint analyses. Cause‐specific hospitalizations and ED visits will be adjudicated by an independent and blinded Events Committee. Adverse ablation procedural events will also be reviewed by the Events Committee. For objective 3 (functional capacity): we are interested in detecting a difference between arms of 3 ml/kg per minute, similar to the typical effect size observed in prior studies, which ranged from 1.6–5.0 (Spertus et al., 2011). A sample size of *N* = 120 (60 per arm) patients with complete peak VO_2_ data at both time points will achieve >90% statistical power to detect a 3 ml/kg per minute (2.78 SD units) difference in the average within‐patient change in peak VO_2_ between treatment arms assuming equal variances using a two‐sided 0.05‐level two‐sample *t*‐test. If the variances of within‐patient changes in peak VO_2_ differ between the 2 arms, the statistical power will decrease somewhat, though remains well above 90% until the variance ratio between the 2 arms increases to a factor of 5. The indicated power is maintained even if 10% of patients have a missing postablation peak VO_2_ data due to death or loss to follow‐up. A similar power analysis was carried out for QoL as measured by the AFEQT. AF ablation was shown to be associated with 23.0 ± 14.4 points improvement in the AFEQT Global Score;(Spertus et al., 2011) with our current sample size we can expect to achieve >90% statistical power for detecting a change in QoL of 9 points (14.35 SD units; > 80% for an 8 point change), allowing for up to 10% of patients with a missing postablation QoL measure.

This trial will enroll approximately 40% females and post‐trial analyses will include confirmation of elevated risk based on gender, and subgroup analyses will evaluate how the response to study procedure varied based on gender. The trial dataset will be available for exploratory analyses while safeguarding the privacy of participants.

## 3 DISCUSSION

The ICM‐REDUCE‐AF is a prospective, randomized, double‐blind, single‐tertiary care center clinical trial that will evaluate whether ICM‐guided early CA intervention for paroxysmal or persistent AF will decrease AF burden, reduce healthcare utilization, and improve functional capacity compared with conventional clinical monitoring and medical therapy. Studies to date have focused on the management of post‐CA atrial arrhythmias based on clinical AF recurrence, which is limited by noncontinuous atrial rhythm monitoring detection and may represent a late presentation of a prolonged course of undetected, subclinical AF. SCAF is common after AF ablation, and its clinical significance still remains controversial. Limited, but compelling recent data indicate a significant association of SCAF with progression to clinically persistent AF, suggesting that early intervention based on SCAF may prevent clinical AF recurrence after CA (Alipour et al., 2017; Pokushalov et al., 2011, 2013). In this study, SCAF is defined as two AF episodes lasting more than 5 min or a single AF episode lasting more than 6 h. Intervening on two AF episodes lasting just more than 5 min may be considered aggressive and re‐initiation of AAD or repeat ablation based solely on the development of SCAF may increase the risk of adverse events. However, we hypothesize that early and proactive treatment of SCAF could improve overall postcatheter AF ablation outcomes.

ICMs provide the opportunity to remotely assess individualized responses to the CA procedure, prior to the development of clinical AF that result in healthcare provider contact. Compared to other wearable technologies such as Apple watches or Fitbits, ICMs are able to provide continuous AF monitoring postcatheter ablation including duration of AF in both symptomatic and asymptomatic patients. Studies have demonstrated that longer maintenance of sinus rhythm after ablation procedure may reverse the underlying adaptive processes, such as electrical and structural remodeling, associated with AF recurrence (Callans et al., 2004; Gerstenfeld et al., 2003; Nanthakumar et al., 2004). Hence, early detection of AF recurrence and the ability to intervene sooner through patient‐triggered remote transmissions before significant left atrial substrate modification can lead to further post ‐AF ablation success. Furthermore, early interventions based on ICM data could have a significant impact on healthcare utilization and cost. We hypothesize that these interventions would decrease the amount of expensive and otherwise preventable use of healthcare resources such as unscheduled clinic, ED visits, and hospitalizations. Nevertheless, it is still possible that early interventions for AF, including changes in AAD therapy and/or repeat CAs, might result in higher healthcare costs. Thus, this important endpoint will be assessed in the current trial.

The ICM‐Reduce‐AF trial seeks to determine whether early clinical intervention based on personalized ICM‐guided data will have any impact on AF burden and clinical outcomes. A positive outcome from this study would lead to a paradigm shift to post‐AF ablation management that will improve clinical and arrhythmic outcomes in an ever‐expanding patient population.

## FUNDING INFORMATION

This study is supported through the National Institutes of Health NIH Grant 1R61HL153001‐01A1.

## CONFLICT OF INTEREST

Dr. David T. Huang is an Editorial Board member of Annals of Noninvasive Electrocardiology and a coauthor of this article. To minimize bias, he was excluded from all editorial decision‐making related to the acceptance of this article for publication. Dr. Wojciech Zareba is the Editor‐in‐Chief of the journal and coauthor of this article. He was excluded from the peer‐review process and all editorial decisions related to the acceptance and publication of this article. Peer review was handled independently by Associate Editor, Dr. Mark Haigney to minimize bias.

## DISCLOSURES

Sinan S. Tankut: None David T. Huang: Research grants from Biosense‐Webster, Medtronic, Biotronik. Fellowship support from Abbott, Boston Scientific, Biotronik, and Medtronic Mehmet K. Aktas: Research grants from Boston Scientific, Medtronic, Biosense‐Webster, Abbott, Astra Zeneca, Consulting fees from Abbott Spencer Z. Rosero: Research grants from Medtronic and Biotronik Wojciech Zareba: Research grants from Boston Scientific, Biotronik, Zoll Inc. Jonathan Steinberg: Research grants from NIH, AliveCor; equity in AliveCor, National Cardiac, BraveHeart; consultant for Medtronic, National Cardiac, BraveHeart, Corfigo, Hillrom, Allergan, Atricure; other with ABIM Jennifer Henchmen: None Robert L. Strawderman: None Ilan Goldenberg: Research grants from Boston Scientific, Zoll, Medtronic, Biosense‐Webster, Biotronik, Abbott, Astra Zeneca

## ETHICAL APPROVAL

A data and safety monitoring board (DSMB) will be convened to independently monitor the conduct and outcomes of the trial. The DSMB will be responsible for monitoring the safety and well‐being of the patients participating in this study and ensuring the ethical conduct of the trial.

## Data Availability

The trial dataset will be available for exploratory analyses, while safeguarding the privacy of participants.
